# Lime finishes: what’s the point? Pressure difference in storm conditions in a changing climate

**DOI:** 10.14324/111.444/ucloe.3623

**Published:** 2026-07-16

**Authors:** Tim Meek, Kate North, C. Douglas Moore, Jens-Arne Subke

**Affiliations:** 1 Biological and Environmental Sciences, The University of Stirling, UK; 2 Forest Research, Northern Research Station, Roslin, Midlothian, UK

**Keywords:** harl, limewash, pressure difference, water penetration, heritage

## Abstract

Climate change is driving international heritage to reconsider the conservation strategies applied to traditional historic buildings. To date, the preferred repair method for rubble stone buildings in the UK has been pointing, and the style employed is the heritage joint: a recessed joint that highlights the profile of the stone with aggregates exposed. This paper challenges that conservation approach, by demonstrating that a bare stone method leaves historic fabric in Scotland vulnerable to water penetration, particularly in the north and west regions where wind-driven rain predominates. In addition, we argue that the heritage joint lacks historic authenticity, and that the accurate presentation of architecture is related to the original functionality of the building system. By turning to the archaeological record, we demonstrate that pre-industrial Scottish vernacular buildings were originally completely lime-coated, and that this was an effective way of managing moisture. By the 18th century, changing tastes expressed a preference for bare stone and ultimately this led to finishes being stripped and the joints between stones being pointed in many different styles, few of which are employed within the heritage sector. The strong winds experienced in storm conditions create external/internal pressure differences on windward elevations. The rain that accompanies wind is attracted into the internal space in these conditions where wall widths diminish, and where there is no external finish. In an innovative experiment, the actualistic pressure differences observed in storm conditions are created using a purpose-built airtight chamber and these pressure differences are accompanied by simulated rainfall. The results of the experiment show that pressure difference is a significant contributor to water penetration at the window embrasure. Crucially, the experiment demonstrates that completely lime coating rubble stone buildings moderates this ingress, while the heritage joint is the least effective barrier to water. The baseline for conserving and improving historic rubble building fabric is considering complete lime finishes as vital tools in building resilience.

## Introduction

The heritage industry recognises the significant challenges involved in adapting traditional pre-industrial rubble buildings to achieve lower thermal conductivity [1–3], improve internal comfort [4] and reduce energy use and running costs [5]. However, far less attention has been paid to whether the original design, functionality and presentation of rubble stone buildings have been accurately understood. This paper examines the impact and subsequent loss of external lime finishes and asks how their reintroduction would moderate water penetration in storm conditions when external/internal pressure differences create an attractive environment for water ingress. While the study is centred on Scotland’s architecture and climate, the results are applicable to other regions where vernacular buildings have been stripped of finishes and where extreme weather events are becoming increasingly frequent.

In response to the energy and climate crisis, Historic Environment Scotland commissioned a PhD research programme at the University of Stirling. As part of this work, a nationwide survey of 311 sites was undertaken to establish the spatial and temporal extent of external lime finishes in Scotland [6]. The survey characterised finish types, thickness and evolution, and was underpinned by detailed desktop analysis of archive material, early photographs, prints, paintings and rare but crucial documentary sources. First published in 2019 and updated in 2022 to include evidence for Scotland’s varying pointing styles, the results were compelling. Survivals of complete finishes were recorded from the Early Mediaeval period (300–1100 CE in Scotland) through to the 20th century in more isolated areas.

Fieldwork observations from this study, together with ongoing research undertaken by Historic Environment Scotland as part of its High Level Masonry Programme, provide evidence for Scotland’s diverse range of complete finishes, pointing methods, changes in building style and the resultant structural flaws. These findings form the baseline for the experimental work presented here. The research demonstrated that the thickness of harl reduced following the introduction of Palladianism in the 18th century [7], and that the use of complete finishes diminished over time. By the late 17th century, ashlar stone had become the preference of the elite, while by the late 18th century, Romanticism promoted the use of split and sawn stone in rustic styles [8,9]. These shifts enabled the development of Scotland’s varied pointing traditions [10], in direct contrast to the universal application of finishes prior to the Long Eighteenth Century (c. 1688–c.1850).

**Figure 1:**
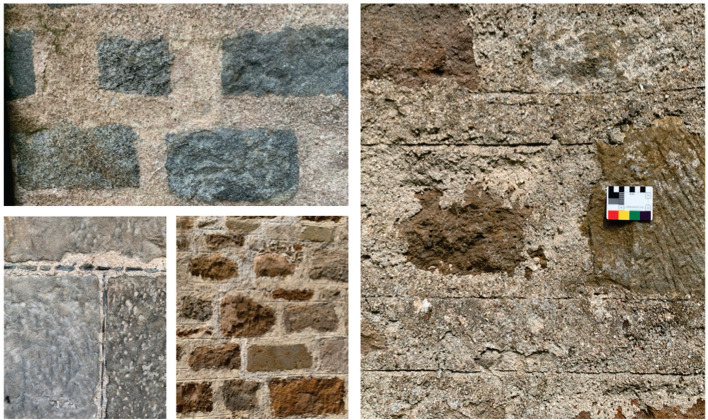
Some examples of the 16 Scottish lime pointing styles c. 18th–19th century.

**Figure 2:**
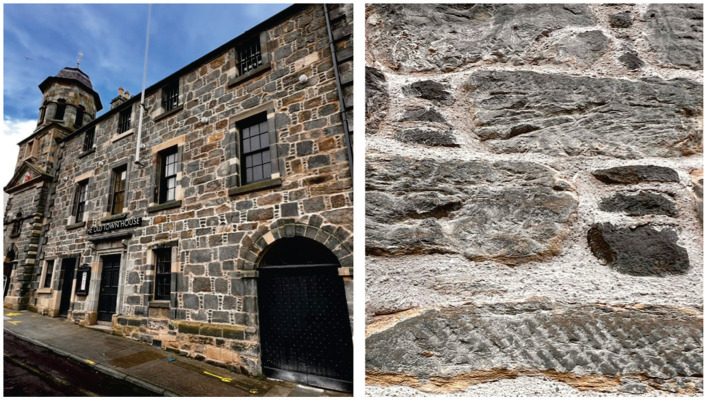
Inverkeithing Town House c. 18th century, an example of the ‘heritage joint’.

Scotland was found to have 16 distinct pointing styles, with the overwhelming commonalities being lining-out: the pressing of mortar while still green, using a purpose-made tool. All the examples were at least flush with the face of the wall and in most examples completely covered the joint (Fig. 1). No evidence was identified in the archaeological record for the now ubiquitous ‘heritage joint’ (Gerard Lynch, personal communication). This method involves filling open joints with new lime mortar and then recessing them with a stiff brush, clearly defining the stone edges and exposing aggregate to produce an artificially aged appearance (Fig. 2). The heritage joint emerged in the early 20th century as a technique for the conservation of ruins and was later adopted for roofed buildings [11], a practice that has continued since.

Further important observations emerged from the fieldwork. Many buildings were found to have been harled or finished during construction [12], a practice that likely aided carbonation – the absorption of atmospheric carbon dioxide to form a durable mortar – demonstrated by the long-term survival of finishes in exposed locations. Less robust limewash was predominantly observed in sheltered areas. Limewash was also seen to extend over harled surfaces and fold around chamfered and roll-moulded details into door and window openings, creating seamless external surfaces.

The fieldwork concluded that there has been a widespread misunderstanding of both the functional and presentational value of rubble stone buildings in Scotland. Prior to the Long Eighteenth Century, all masonry surfaces were coated with harl or plaster. This finding challenges an embedded historiography that has directly informed practical conservation approaches [13,14].

The aim of this paper is to highlight an inherent flaw in the design and construction of Long Eighteenth-Century residential buildings and to examine the role of lime finishes in mitigating the resultant water penetration. In doing so, it demonstrates the inappropriateness of the heritage joint as a conservation methodology. The focus is on the reduced wall width below the windowsill, a repeated construction detail driven by the pursuit of classical symmetry. This change in practice created a vulnerable transverse joint beneath the sill and within the supporting masonry below, which, as the archaeology demonstrates, was managed by lime coats. These coats were crucial because during storm conditions, the velocity of wind is curtailed abruptly at the wall face, generating a pressure difference between the external and internal environments, and the difference in pressure creates an attractive internal environment for moisture in adverse conditions.

To investigate this mechanism, a purpose-built wall with a window opening – normally used for building-craft training at the Scottish Lime Centre Trust (SLCT), Charlestown, Fife, KY11 3EN (National Grid Reference: NT 06478 83813) – was employed. A timber airtight chamber was constructed within the window embrasure. External and internal pressure differences were replicated by extracting air from the chamber while applying simulated rainfall representative of storm conditions. Water ingress was quantified at 10 Pa pressure-difference increments. In response to the resulting inundation, a range of Scottish lime finishes was applied and their moderating influence assessed, before being directly compared with the performance of the heritage joint.

### Wall morphology – the reduction in wall width and the resultant points of vulnerability

**Figure 3:**
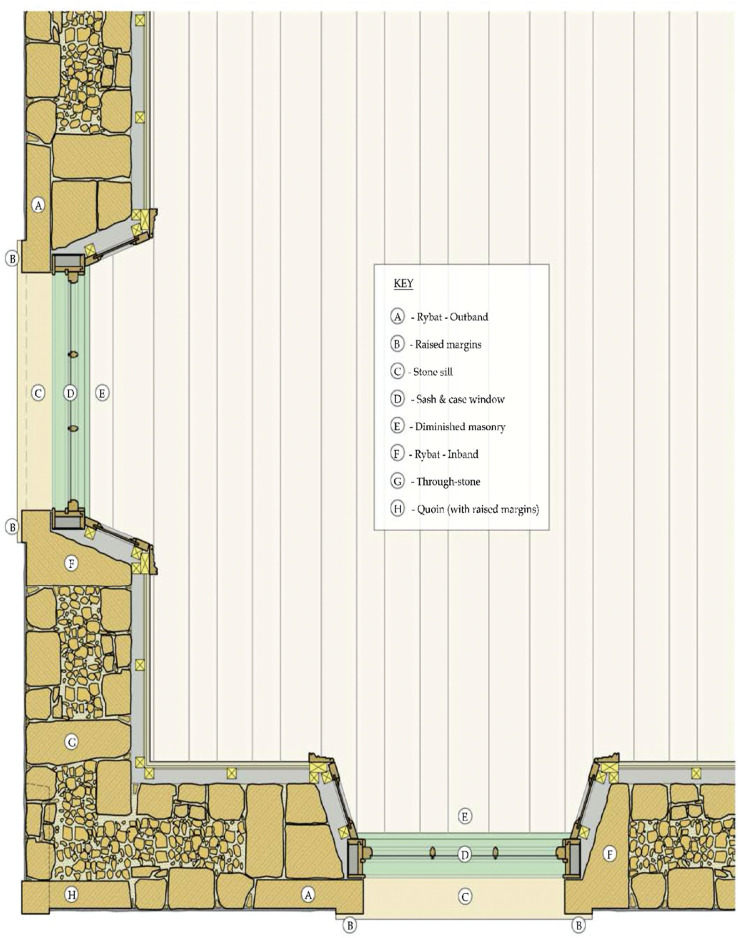
Schematic plan showing a typical wall of the Long Eighteenth Century.

**Figure 4:**
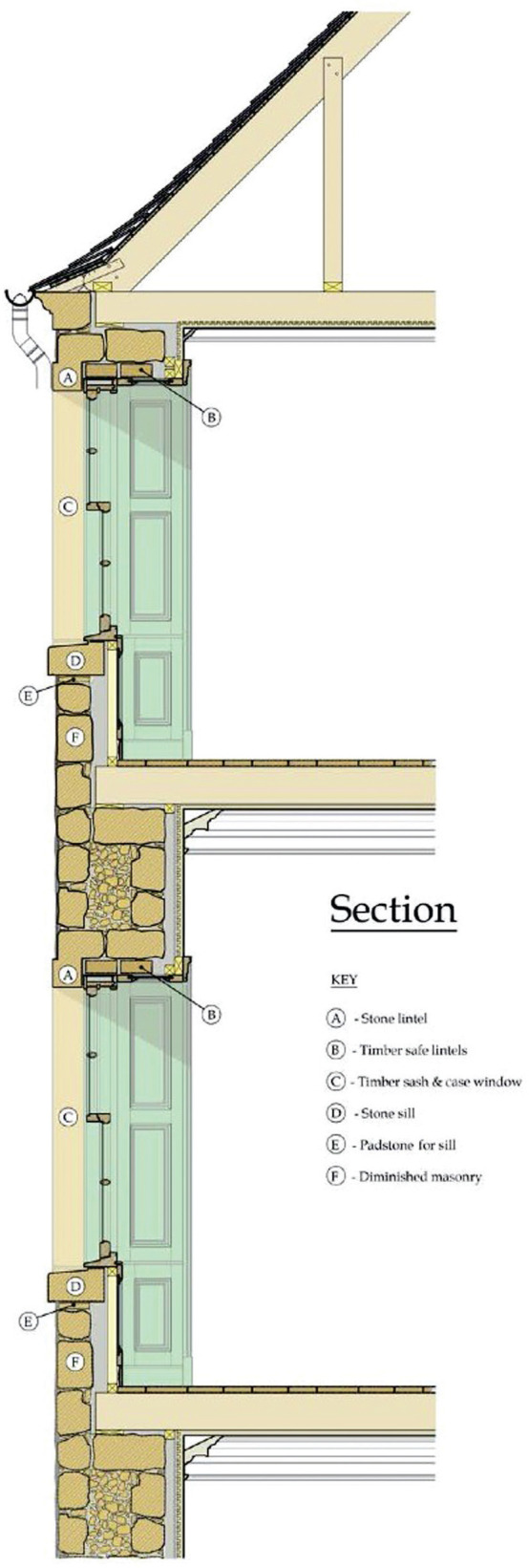
Schematic section of a typical wall of the Long Eighteenth Century.

In tandem with the evolution of finishes, the survey noted that with the arrival of neo-classism in Scotland in the late 17th century, wall widths generally reduced from approximately 1.5 m to 600 mm (Fig. 3). In key areas, such as windowsills and lintels, masonry could be as narrow in section as 150 mm (Fig. 4). It was also noted that the number and size of windows increased throughout the period of the Long Eighteenth Century, sometimes accounting for approximately one third of the total volume of the elevation. This is problematic, particularly where multiple window openings existed on two separate but adjacent corners. By combining the visualisations of the section and plan it is possible to understand the profile of walls as a series of substantial piers of approximately 600 mm width, triangulated at the corners and linked by insubstantial ties approximately 150–200 mm wide under the windows. This design was universal within the context of domestic architecture.

**Figure 5:**
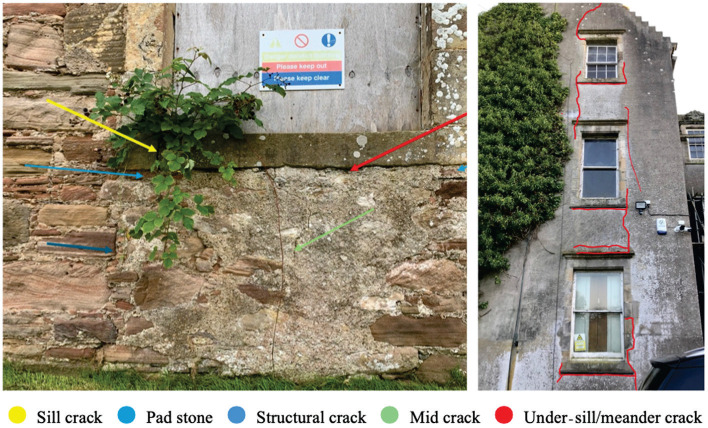
Examples of the cracking that occurs in and around the under-sill area, extending from ground level to crowsteps.

**Figure 6:**
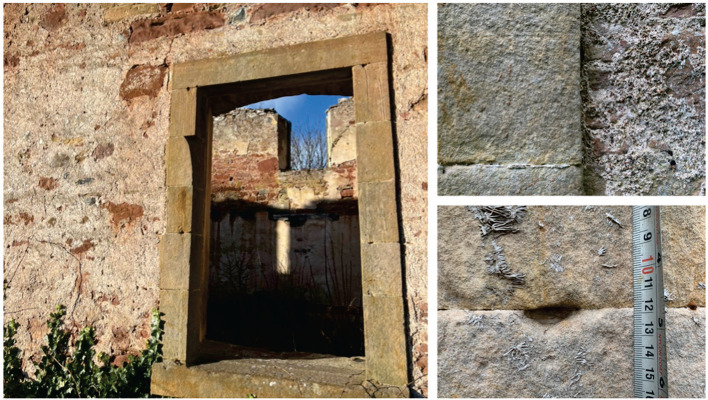
Left: An example of a raised margin and the relationship between the sill and the lintel. Right: Examples of the fineness of the joints between each of the stones that form the margin.

**Figure 7:**
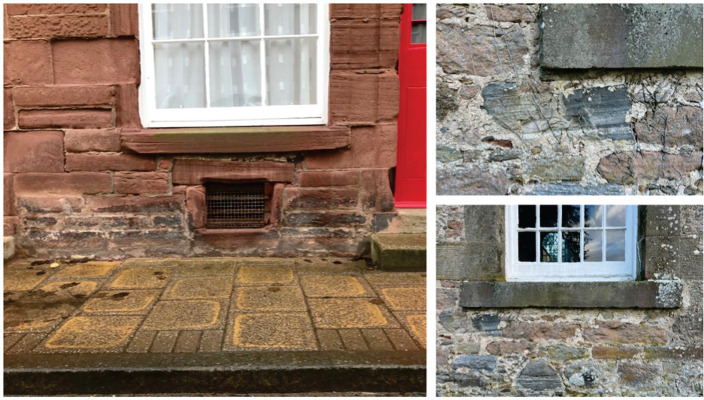
Examples of how the sill was supported on padstones.

A common observation during the survey was cracking of the external envelope around the windows of historic buildings that often extended from ground level up through the masonry, sills to crowsteps, skews and eaves tabling (Fig. 5). This phenomenon frequently occurred when raised margins with very fine joints and little tolerance for adjustment were introduced in the late 17th century (Fig. 6). Masons were required to place the sill on padstones located to the left and right of the window opening, to ensure that the sill was placed and remained level and in the exact plane of the wall necessary to accommodate the margins. The padstones might have been as small as a single piece of slate or a larger profiled section of sandstone (Fig. 7). The accumulated compressive load of the margins was therefore transferred to the padstones. In this scenario, the under-sill joint and the masonry below the sill was not under compression, because the rubble stone was slotted in from the face of the wall, rather than built from above. The under-sill area was especially vulnerable to water inundation because of the greater discharge from window glass. Both the sill and lintel have a transverse joint, being any joint in a single skin of masonry without a core and inner leaf. This is problematic because British Standard (BS) BS5628-3:2005 – a code of practice for the use of masonry, specifically focusing on materials and components, design and workmanship – makes clear that in no circumstances may a wall of 190 mm or less be left exposed, even in the most benign of conditions. Working within the code’s parameters, single-skin masonry would be regarded as an unsuitable construction without a coating. In short, the heritage industry expects once-coated, often deteriorated fabric in exposed conditions to perform like modern masonry with vapour barriers and render.

### Wind-driven rain and the impact of internal/external pressure differences

Scotland has some of the highest rainfall and wind speeds in the UK [15]. Spells of intense rain with a driving component are becoming more common and the relationship between the width of masonry units and a deteriorating climate have become crucial. Assessments have been made that examine the impact of wind-driven rain (WDR) upon building fabric [16–18]. The presumption in this work and others is that WDR is the agency that facilitates water penetration, as well as the capillarity of different stone and joint types [19]. This paper accepts the importance of WDR and capillarity. However, wind also creates pressure differences between the outer face of the windward elevation and the interior space, and that pressure difference between the two environments is also an impactful contributor to inundation within the context of non-airtight historic structures. Wind is the corollary of pressure difference (PD), and the experiment below compares the capacity of lime-harled and uncovered masonry surfaces to resist air and water penetration in a variable air pressure environment where wall thickness diminishes.

Furthermore, air distributed around a building may have differences in pressure. These differences are related to three primary factors: the geometry of the structure, the angularity of any additional features, and the height of a building and the roof pitch. Wind speed and its direction also influence air pressure differences. However, when wind direction is in line with a specific elevation, the pressure at that point is defined by:

P=Kpov   o22,

where *P* is the elevation pressure point in Pa, *K* is the drag coefficient, *vo* is the wind velocity in metres per second and *p_o_* the force of wind velocity in kilograms per square metre [20].

The air flow that envelopes a structure, rather than confronting a vertical face, does so as a dynamic flow with static pressure [21,22]. However, when low pressure air with an accompanying wind confronts the vertical face of the building, the wind velocity is curtailed, and stagnation pressure is created as the wind and its inherent kinetic energy [23] decelerates. The stagnation point denotes the height on a building elevation at which the wind is at its greatest velocity when it contacts the vertical face and divides around the sides and roof. In addition, complex building morphology – shape, height, sculpture, castellated parapet and roof pitch, for instance – determines the local pressure coefficient at any given point. The net pressure varies significantly around the building, from positive pressure on the windward side, to negative pressure on the leeward side. It is significant that the stagnation pressure point is in the upper area, because the evidence of water penetration in the upper area is ipso facto likely to be apparent lower down the elevation due to the forces of gravity. Identifying sources of penetration can be difficult, but single-skin, diminished masonry areas are obvious starting points in any assessment.

**Figure 8:**
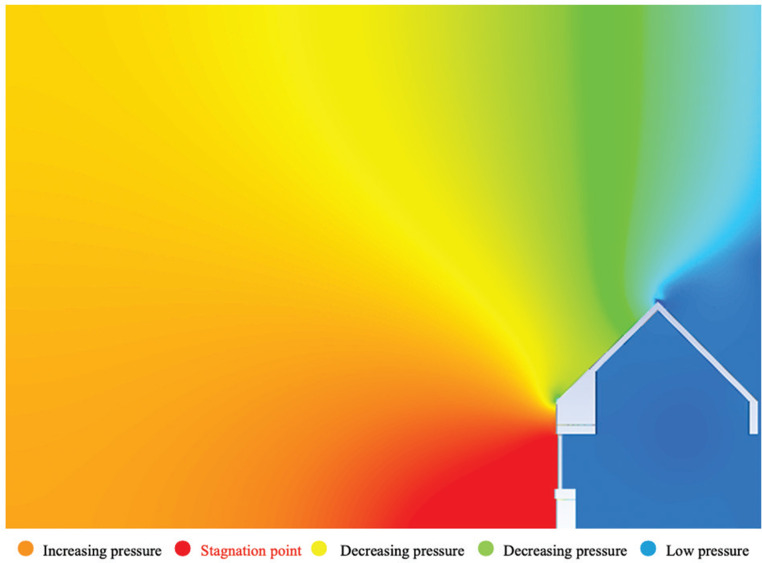
Schematic notional building facing southwest, describing pressure differences relative to changes in the building geometry.

Air infiltrates and exits the historic building envelope through several places, mainly the roof, eaves, doors and windows [24]. Cracks in masonry may form additional ‘air leakage paths’ [25,26]. These cracks may be small – almost too small to be seen – but these can be the source of inundation in storm conditions where external pressure is greater than internal pressure (Fig. 8). Figure 8 illustrates a notional building facing southwest, with an inline directional wind, and a 45° pitched roof. The use of the illustration reflects the model used in the experiment below and is not described with a scale. Within the illustration, increasing positive pressure is indicated by orange, and stagnation pressure is indicated by red. Variable pressure on the roof is dependent on angle and indicated by yellow and green. Negative pressure on the leeward side and internal space is indicated by blue. Positive external and negative internal pressure differences are at their greatest in storm conditions when air infiltration on the windward elevation is less than the sum of air exfiltration on the side and leeward elevations [27–32]. During storms Ciara (8–9 February 2020) and Aiden (1–2 November 2020), gusting pressure differences of between c. 90 and 280 Pa were recorded within a roofed structure in Stirlingshire (Meek, personal observations), with wind speeds of between 70 and 80 mph and rainfall 100–177 mm in a 24-h period [33].

### The aim of the experiment

The aim of the experiment was three-fold: first, to replicate actualistic environments where pressure is exerted on the windward elevation, and where there is uncontrolled air leakage from all other elevations and the roof combined; second, to critically evaluate the prevailing conservation practice of using recessed pointing with exposed aggregates – commonly referred to as the ‘heritage joint’; and third, the results of the evaluation were compared with traditional Scottish lime coats. To test the impact of different pointing and coating methods on moisture resistance, we conducted a controlled experiment simulating storm-driven water ingress. Specifically, we measured water permeability across three conditions: (a) a 465 × 0.065 mm under-sill crack, (b) an intact under-sill finished with high-calcium hot-mixed mortar and (c) natural hydraulic lime mortars.

## Methodology

**Figure 9:**
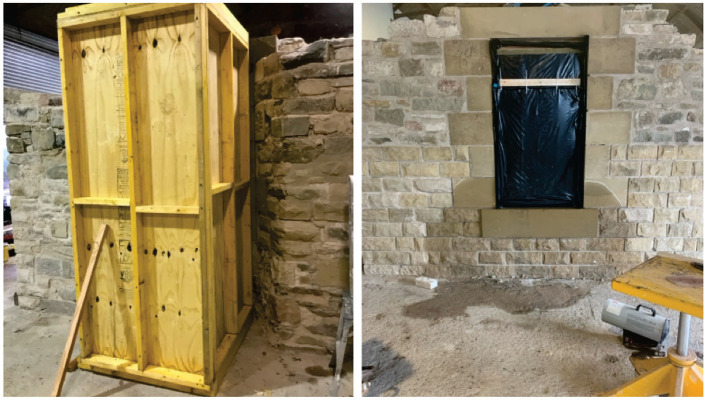
Left: The rear, ‘interior’ of the wall located within the window embrasure. Right: The ‘exterior’ of the wall.

An experimental sealed chamber was constructed off site, then installed within a simulated window embrasure constructed of Dunaverig Old Red Sandstone wall measuring height (H) 2.3 m × width (W) 0.6 m at the SLCT. The total wall length was 3.6 m. A pressure difference across the window embrasure was created by extracting air from the chamber to create specific internal/external pressure differentials in accordance with the British Standard BS EN 13829-2001 ‘Thermal performance of buildings – determination of air permeability of buildings – fan pressurization method’. The method was scaled down and substituted with an industrial dust extractor, which recreated the pressure differences defined in the British Standard above. The construction and installation of the chamber, and all the building and associated lime work, were undertaken by the first author, who is suitably qualified and experienced. During construction, the side, floor and roof panels of the chamber were glued and screwed together. Prior to installation, the masonry was tooled flush internally to accommodate the chamber. The open face of the chamber was scribed to the profile of flattened masonry. Discrepancies between the open face of the chamber and masonry were subsequently filled with polyurethane expanding foam to ensure an airtight fit between the wall and the chamber (Fig. 9).

### Creating pressure difference, monitoring and pressure regulation

The current Building Regulations (approved document Part L1A 2010) recognise the phenomenon of air leakage within the context of new building and requires that a new dwelling should achieve an air leakage of 5 m^3^/hm^2^ or less, using a method known as the ‘blower door test’ (BDT). This tests that a new building is constructed to a recognised standard of air exchanges and must comply with British Standard BS EN 13829:2001 ‘Thermal performance of buildings – determination of air permeability of buildings – fan pressurisation method’. This is the currently accepted UK maximum pressure difference using the BDT as 50 Pa. Recognising the impact of more extreme weather events, the United States employs the American Society for Testing Materials (ASTM) standard for testing air leakage and has increased the BDT value to 75 Pa. In this experiment we downscaled the baseline value to 70 Pa. This value is considerably under the real pressure difference of 290 Pa recorded during Storm Aiden (1–2 November 2020, previously mentioned).

**Figure 10:**
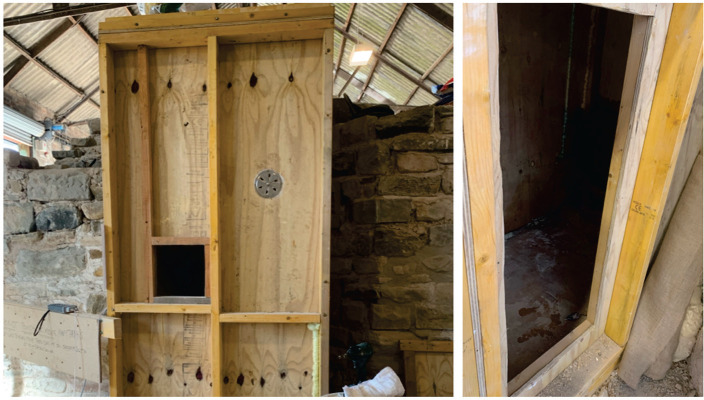
Left: The rear of the chamber with glazed viewing port, sliding vent. Right: Access to the chamber via demountable side panel.

The downscaled BDT pressure difference was created using an SIP 1.5 industrial dust extractor, collection volume 183 m/h, motor: 230 V~50 Hz 1100 W (SIP Industrial Products, Shepshed, Loughborough, UK). This was permanently housed on one of the sides of the chamber (rear left) and fenced off for the duration of the experiments. The pressure was monitored with a calibrated digital manometer (Comark C9500, Fluke Precision Measurements Ltd, Norwich, UK), to assess actual pressure differences between the external environment and the interior of the chamber. The manometer expressed pressure differentials at 10 Pa intervals. The pressure was regulated within the chamber using a sliding vent that reduced or increased the pressure difference, dependant on the differences required, and the prevailing atmospheric conditions within the SLCT workshop. The chamber was provided with a glazed port at the rear for in-trial observations and a demountable side panel for access between pressurised runs (Fig. 10).

### ‘Window’ design and construction

The simulated window embrasure on the ‘windward’ side of the experimental sealed chamber was closed with a timber frame covered in ply and then a secondary sheet of damp proof membrane. The panel was carefully scribed into the window opening to ensure a tight fit. The objective of covering the panel with membrane was to mimic the discharge of water from glass in an intense storm scenario. The window area was H 1.675 × W 0.9 m and the area under the sill was H 0.46 × W 1.25 m, with a wall width under the sill of approximately 0.190 mm.

### ‘Rainwater’ delivery system

Increased intense storms with high volumes or rainfall have been recorded over the last decade, with the highest rainfall in January 2015 peaking at 341 mm at the Honister Pass, Cumbria (Met Office). The objective of the rainfall simulation was to provide a realistic volume of water. An independent hose system, using an off-the-shelf garden irrigation system from the tap to the window, recreated a high rainfall episode. The water from the tap was restricted to 1.5 bar with the volume of water regulated at 2.78 L/min, which is comparable with Storm Desmond in 2015.

### Water capture

Water was captured on the ‘inward’ face of the wall in a fixed trough made of galvanised steel with stopped ends and fitted to the floor of the chamber at 60 mm from the face of the wall. Water retention pads with a capacity of 340 mL, made from a combination of paper pulp and a propriety ‘super-absorbent’ polymer, were placed in the trough. Pads were weighed prior to each run and the combined weight of ‘dry’ pads was deducted from the combined weight of the pads/mat with water. The pads were placed and retrieved from the trough via the demountable side panel between each pressured run and subsequently weighed.

### Construction mortar

Two separate experiments were run using different mortar mixtures, applied sequentially and employing the same wall. The mortar used in Experiment 1 replicated the construction mortar undertaken by the SLCT. It was made with Melville Gates (Angle Park, Ladybank, Cupar, Fife, Scotland) coarse sand and Natural Hydraulic Lime (NHL) to reflect the local hydraulic limes historically quarried and manufactured at Limekilns (Fife, Scotland). The hydraulic lime used in the experiment was manufactured by St. Astier and classified as compressive strength 2 MPa. The mortar used in Experiment 2 was a hot mixed mortar (HMM), meaning the sand and aggregates were combined with quicklime and sufficient water to produce an exothermic reaction. Melville Gates sand and Shap high calcium kibbled quicklime at a rate of 3 parts sand and aggregates to 1 part quicklime were combined by volume. On slaking, this produced a mortar with a binder ratio of c. 2:1 that closely resembles the high binder to sand aggregate ratios found in historic buildings [34].

The experimental stages were conducted sequentially. Experiment 1 (NHL mortar): at each stage – pointing, sneck harl (meaning a partial harl dashed only over the joints), harl and limewash – a three-month interval was left between the applications. Staff at the SLCT undertook the curing regime in accordance with best practice. The SLCT is a recognised lime training provider. The HMM experiment intervals were extended to six months, which better reflects the longer time required for a high calcium mortar to carbonate.

In both experiments, the term ‘open slot’ refers to the experimental wall with a simulated constructional crack, formed by sawing through the under-sill joint with a hacksaw blade < 1 mm, formed by filing down the blade. The term ‘closed slot’ refers to a wall with a simulated crack sealed, and the wall pointed employing the heritage joint.

### Harl and sneck harl applications

**Figure 11:**
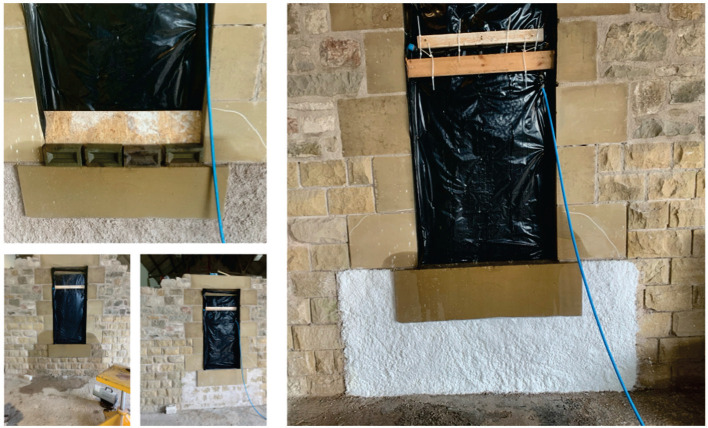
Top left: Under-sill area with harl. Right: harl and limewash. Lower central: Sneck harl application. Lower left: The heritage joint application.

Harl was applied in two coats at c. 10 mm, with sufficient time between applications for the mortar to ‘firm up’, but remain ‘leathery’, meaning that it was no longer a semi-liquid but still malleable enough to mould. The sneck harl was applied as a single coat of c. 10 mm thickness (Fig. 11).

### The experiments

The experiments examined water penetration through single-skin masonry below a window in two scenarios: the first being a wall constructed and finished with NHL mortars, which was then contrasted with the second, HMM alternative. Timings for individual runs were relative to the wall condition: open slot, closed slot, sneck harled, harled, and harled and limewashed. The open slot was tested at 2-min intervals, alternating between 0 and 10 Pa, to 0–70 Pa with six repeats for each 10 Pa increment. The closed slot was tested at 30-min alternating pressure differences. Sneck harled, harled and harled, and limewashed scenarios were tested at 60-min alternating pressure differences.

### Statistical analysis

The impact of air pressure difference and wall covering treatment on water ingress was statistically analysed in the R Program for Statistical Computing version 4.3.2 (R Core Team, 2022). A generalised linear model (GLM) was used, assuming a gamma error distribution (GLM function in the stats package). We were not interested in the interaction between air pressure and treatment, and this was therefore excluded from the model, giving a final model (water ingress ~ treatment + pressure difference). Post hoc pairwise comparisons between treatments were conducted using Tukey’s honestly significant difference (HSD) test (lsmeans function in the lsmeans package).

## Results

Increasing air pressure also increased the volume of water penetrating the wall in a statistically significant way (GLM: X^2^_1,250_ = 5053, *p* < 0.001). Differences in water penetration were noted at low pressure differences with an open slot. In the NHL trial at 0 Pa difference, the rate of penetration was around 120 mL/min, increasing to around 380 mL/min at 70 Pa PD. In the HMM trial, water increased from 120 mL/min to 460 mL/min over the same pressure range. In both the NHL and HMM scenarios, the impact of pressure difference at 70 Pa is significant enough to be visualised through the viewing port. The presence and type of surface finish had a highly significant influence on the volume of water penetration (GLM: X^2^_7,250_ = 110, *p* < 0.001), and the amount of water penetration was statistically significantly different between all lime treatments applied (Tukey’s HSD: *p* < 0.05). Water inundation was progressively reduced when the differing lime treatments were applied (Fig. 11).

With the slot filled, it was no longer possible to visually observe the increased flow of water penetration, even at 70 Pa, but the weight of the pads indicated that increased volumes were related to PD. By accessing the chamber during PD runs and observing the wall at floor level, it was possible to see water bubbling in the perpendicular joints at 70 Pa. This significant observation correlates with the difference in compression between bedding and perpendicular joints.

**Figure 12:**
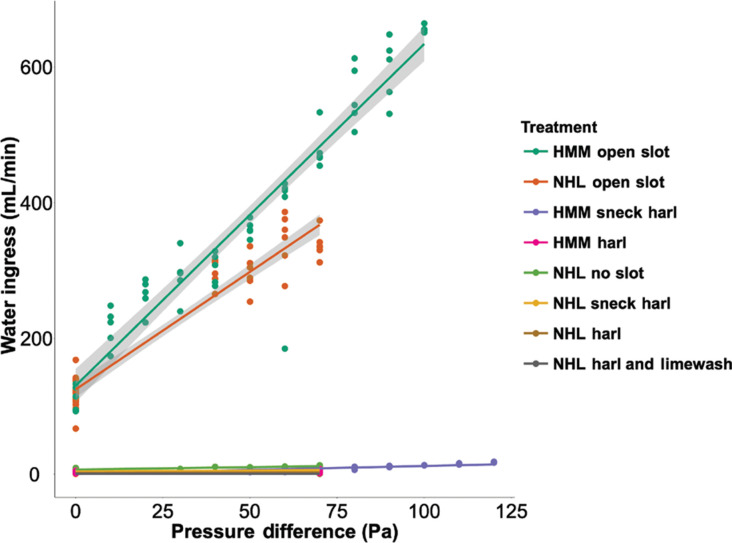
Water entering the chamber with NHL and HMM in an open slot scenario. Contrasting harl scenarios are also shown for comparison. Lines indicate linear regressions. The shaded areas represent the standard error of the mean. Lines indicate linear regressions.

**Figure 13:**
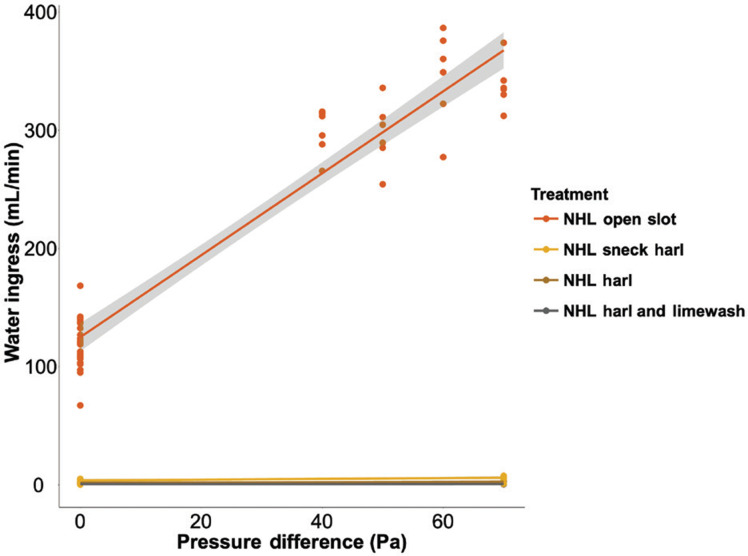
Water ingress in an NHL wall with an open slot relative to pressure difference and the moderating response of NHL mortars.

**Figure 14:**
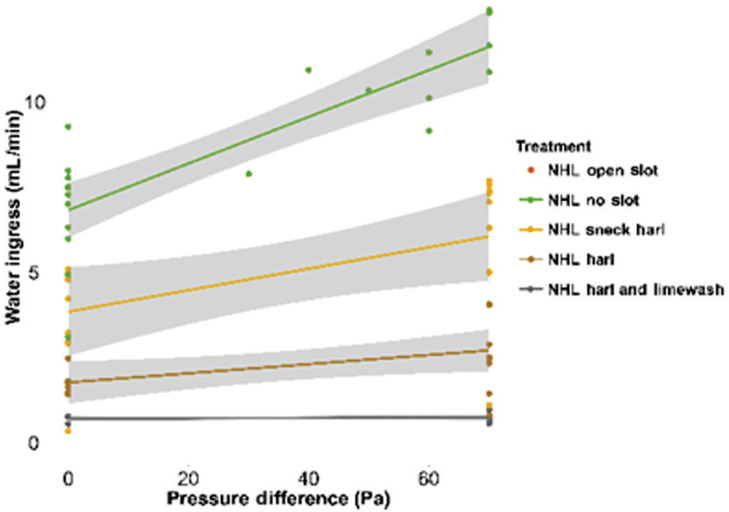
Water ingress for different surface covers and across pressure differential. Data are identical to those in Fig. 4, but scaled to resolve low water ingress values with the varying treatments.

**Figure 15:**
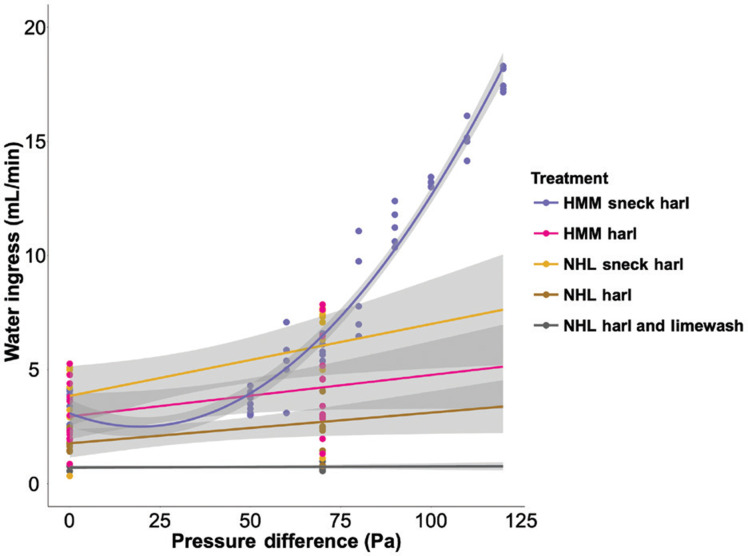
Water ingress values for NHL and HMM treatments compare water ingress between the two mortar types. The HMM Sneck harl data series displays an exponential increase.

In the NHL pointing trial the penetration rate at 0 Pa difference was 7 mL/min, increasing to 12 mL/min at 70 Pa PD (Fig. 12). In the NHL sneck harl trial at 0 Pa difference, the rate of penetration was 4 mL/min, increasing to 7 mL/min at 70 Pa, an increase of 3 mL/min. In the HMM trial at 0 Pa, the rate of penetration was 2 mL/min, whilst at 70 Pa this increased to 7 mL/min, an increase of 5 mL/min (Figs. 13 and 14). In the NHL harl trial at 0 Pa difference, the rate of penetration was 2 mL/min, increasing to 3 mL/min at 70 PA PD. This compares to the HMM trial of 3 mL/min versus 5 mL/min for the same pressure change. In the NHL harl and limewash trial, as well as the HMM trial, there was no measurable increase in water ingress across the full pressure range. When the pressure difference was raised to 120 Pa (HMM), there was a significant exponential rise in inundation, which was particularly noticeable during the sneck harl model, rising from 2 mL/min to 14 mL/min. Full harl showed a lower increase from 3 mL/min to 5 mL/min (Fig. 15).

## Discussion

The anomaly in the greater volume of water entering the chamber with HMM harl compared with NHL harl is counter-intuitive, given the current understanding of the benefits of hot-mixed mortars [35], but can be explained by the observations made before and during the pressurised runs. High calcium, high binder content mortars are prone to shrinkage as they cure [36], and if the mortar remains ‘plastic’ the process of shrinkage continues. The HMM mortars showed visible shrinkage cracks at all stages: building, sneck harl and harl.

**Figure 16:**
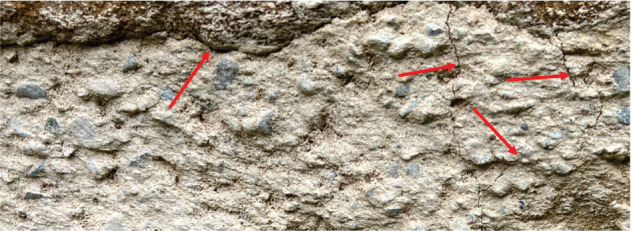
HMM shrinkage cracks indicated by the red arrows.

In the NHL trial, shrinkage cracking was not visible and only became apparent when bubbling water at the perpendicular joints was seen during the pressurised trial from within the chamber. This stopped when the sneck harl was applied. The bubbling remained evident with the HMM at all stages until the harl was limewashed, at a pressure difference of 70 Pa and above, after which no measurable water entered the chamber. HMM shrinkage can be viewed as a contributor to water penetration within this context, which was resolved only when limewash filled the cracks (Fig. 16). The relatively poor performing NHL sneck harl may be attributed to changes in the build quality of under-sill masonry. The experiment inherited the wall, but taking down and rebuilding the under-sill area for the HMM trials was undertaken by the first author.

During the open slot stage of the experiment, two fans were introduced to replicate the impact of WDR. The fans were placed on the ‘outside’ of the wall driving ‘rain’ back into the window opening, sill and under-sill masonry. This had no impact on the volume of water entering the chamber – only when a pressure difference was introduced was there any increase.

Another significant observation within the study was the condition of harl and limewash when the pressure testing regime was concluded. While the HMM mortar and wash remained largely complete, they were damaged and friable. Had this been exposed and not protected, it is unlikely to have survived extreme wind or frost damage. Conversely the NHL mortars remained robust and without damage with only slight degradation to the limewash.

As this paper demonstrates, there are vulnerabilities in reducing wall widths between windows which are clearly visible, often from ground level and travelling up through the structure. Given that these represent movement, it is reasonable to assume that there will be smaller invisible cracking taking place. The water bubbles observed within the chamber are illustrative of the micro-cracking expected when buildings make imperceptible shifts.

## Conclusions

The fieldwork on which this experiment is predicated demonstrates that there has been an underestimation of the extent of lime finishes in Scotland with no previous understanding of how they evolve into the various pointing styles. While the study has focused on Scottish traditional buildings, vernacular buildings anywhere in the UK that have been stripped of their finishes are vulnerable, particularly where wind and rainfall are high. Moreover, the experimental work demonstrates that anywhere in the world that experiences high winds and rain would benefit from the research. We created realistic external/internal pressure differences at well below those real pressure differences experienced in storm conditions, namely storms Aiden and Ciara. The key findings of the study are:

1. External/internal pressure difference is a major contributor to water inundation anywhere where wall widths diminish and where cracking has occurred, however small and irrespective of mortar type. There was a simple correlation between the volume of water entering the chamber and increased pressure difference. This was particularly marked in pressure differences of more than 30 Pa and exponential when exceeding the 70 Pa baseline employed for the bulk of the experiments. Innovative experimental testing therefore reveals that extreme weather amplifies pressure differentials that drive moisture into masonry.
2. The impact of pressure difference can be regulated by successively increasing the depth of coverage of lime coats, the most effective cover being full harl and limewash, the least effective the heritage joint, irrespective of binder type.
3. Notwithstanding the statement above, the NHL mortars proved to be significantly more durable and less prone to shrinkage at all stages. The experiment also revealed that the initial tamping of the HMM is an inadequate measure to stop longer-term shrinkage, because the mortars are not ‘fixed’ until fully carbonated.
4. To date there is little to compel building owners, local authorities, national amenity societies and statutory advisors to recoat historically lime finished buildings, even when the evidence from archaeological and desktop studies is compelling. There are no lime training centres in Scotland teaching anything other than the heritage joint. This experimental work brings together the cultural and physical evidence helping to inform policy which is necessary because there is a widespread misunderstanding of the role of finishes – lime finishes ensure that rubble buildings are seamless and less vulnerable to the impacts of WDR and pressure differences.

## Data Availability

All data generated or analysed during this study are included in this published article.
